# A Wearable System Based on Multiple Magnetic and Inertial Measurement Units for Spine Mobility Assessment: A Reliability Study for the Evaluation of Ankylosing Spondylitis

**DOI:** 10.3390/s22041332

**Published:** 2022-02-10

**Authors:** Adriana Martínez-Hernández, Juan S. Perez-Lomelí, Ruben Burgos-Vargas, Miguel A. Padilla-Castañeda

**Affiliations:** 1 Applied Science and Technology Institute (ICAT), National Autonomous University of Mexico (UNAM), Mexico City 04510, Mexico; adriana.mah@outlook.com (A.M.-H.); salvador.perez@icat.unam.mx (J.S.P.-L.); 2 Rheumatology Service Unit, General Hospital of Mexico “Dr. Eduardo Liceaga”, Mexico City 06720, Mexico; r.burgos.vargas@gmail.com

**Keywords:** spine, inertial measurement units, Kalman filter, human movement, ankylosing spondylitis

## Abstract

Spinal mobility assessment is essential for the diagnostic of patients with ankylosing spondylitis. BASMI is a routine clinical evaluation of the spine; its measurements are made with goniometers and tape measures, implying systematic errors, subjectivity, and low sensitivity. Therefore, it is crucial to develop better mobility assessment methods. The design, implementation, and evaluation of a novel system for assessing the entire spine’s motion are presented. It consists of 16 magnetic and inertial measurement units (MIMUs) communicated wirelessly with a computer. The system evaluates the patient’s movements by implementing a sensor fusion of the triaxial gyroscope, accelerometer, and magnetometer signals using a Kalman filter. Fifteen healthy participants were assessed with the system through six movements involving the entire spine to calculate continuous kinematics and maximum range of motion (RoM). The intrarater reliability was computed over the observed RoM, showing excellent reliability levels (intraclass correlation >0.9) in five of the six movements. The results demonstrate the feasibility of the system for further clinical studies with patients. The system has the potential to improve the BASMI method. To the best of our knowledge, our system involves the highest number of sensors, thus providing more objective information than current similar systems.

## 1. Introduction

Ankylosing spondylitis (AS) is among the 200 specific disorders comprising rheumatic diseases, which are some of the leading causes of morbidity in the world. AS mainly affects the spine, the sacroiliac joint, and entheses [1]. AS is a chronic degenerative disease that begins with inflammatory back pain and severe inflammation of the sacroiliac joints. In more advanced stages, it can also affect the cervical spine. Chronic inflammation of the axial joints generates bony bridges, called syndesmophytes, which can join two vertebrae, limiting the movement of the spine and, in more severe states, limiting lung capacity [2,3].

The prevalence of AS in the Mexican population is around 0.6% to 0.9% [4]. Burgos-Vargas et al. [5] reported that the incidence of new cases of AS in their study of the Mexican population occurred mainly in young adults between 15 and 30 years. Despite the prevalence and the importance of the affected musculoskeletal structures, its diagnosis can be delayed by 3–11 years [6]; the Assessment of Spondyloarthritis International Society (ASAS) has designed evaluation and diagnostic criteria for patients with high suspicion [7]. The current medical tests include (a) blood tests, (b) medical imaging (CAT and MRI), (c) inflammatory activity (BASDAI index), (d) functional capacity (BASFI index), and (e) metrology (Bath Ankylosing Spondylitis Metrology Index “BASMI”).

**Figure 1:**
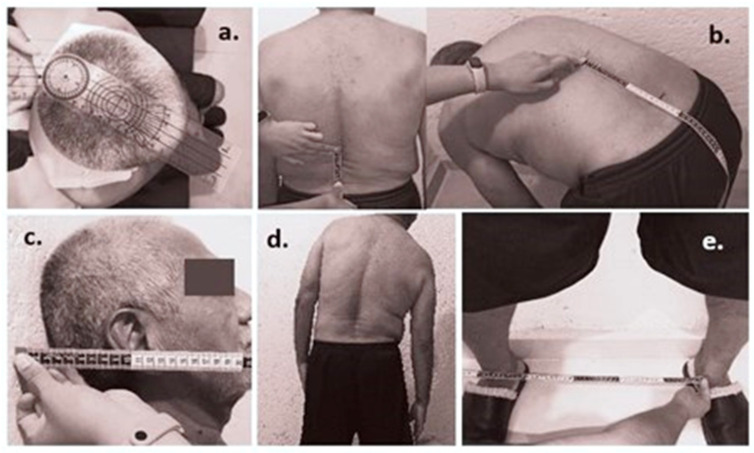
BASMI measurements: (**a**) cervical rotation (observed from a view perspective above of the subject head); (**b**) modified Schober test; (**c**) tragus-to-wall distance; (**d**) lumbar side flexion; (**e**) intermalleolar distance.

The BASMI correlates with the patients’ functional disability and includes a series of five parameters to assess the spine and hip mobility. The measures contemplate cervical rotation, tragus-to-wall distance, lumbar side flexion, modified Schober Test, and intermalleolar distance (Figure 1) [8].

Mobility assessment of the spinal joint and the sacroiliac articulations is essential for diagnosis and evaluation of the patient’s state. However, medical experts apply the BASMI index using a measuring tape and goniometers to obtain the measurements. Thus, systematic and subjective errors can appear as a function of medical experience, the correct use of the instruments mentioned above, erratic or compensatory movements of patients, or observation errors. All of these factors cause the BASMI measurements to lack accuracy and repeatability, according to some studies of the current clinical assessment [9,10,11].

In addition, the use of measuring tapes and goniometers makes the BASMI lose sensitivity to changes in the patient’s status and progress; on the other hand, the spine and the sacroiliac joints generate complex movements, which cannot be evaluated with the BASMI method. Therefore, it is important to research and develop new technology-based mobility assessment methods to extensively assess the joints directly with adequate precision and repeatability, along with sensitivity to changes in information.

Optical marker-based systems have been the most widely used for tracking motion and evaluating spinal mobility [12,13]. Garrido-Castro et al. [14] established a new index (UCOASMI) correlating BASMI with optical measurements. Even though these systems have shown great accuracy and reliability, they are expensive, viable only indoors, and need a complex setup with external sources such as cameras and spaces explicitly conditioned for these purposes, limiting their use in standard clinical conditions.

In recent years, magnetic and inertial measurement unit (MIMU)-based systems have gained attention for human motion analysis. MIMUs have been applied in several clinical areas such as gait tracking [15], fall-risk detection [16], upper-limb human motion [17], diseases in the lower limb [18,19], rehabilitation [20,21], sports [22], spinal loads [23], and ergonomics [24]. Specifically, few studies that showed the feasibility of applying these systems in spinal mobility assessment have been reported [25,26].

MIMU-based systems for spine mobility assessment present some advantages; they are completely self-contained, allow ambulatory tracking, and can be unobtrusively attached to the patient’s spine. Unlike traditional assessing methods, MIMUs can measure the continuous variation of angles in dynamic movements and maximum range of motion [27,28]. Some works studied the accuracy of MIMU-based systems, correlating them with optical systems; they showed a high level of agreement between methods and greater feasibility of MIMU systems in clinical practice [29,30,31].

Recently, some studies assessed AS patients using MIMU systems. Aranda-Varela et al. [31] established the IUCOASMI index, correlating the BASMI with their obtained metrics (flexion/extension, lateral flexion, and rotation at lumbar and cervical region) using two ViMove brand inertial sensors. O’Grady et al. [32] used two ViMove sensors to evaluate the reliability in measuring spinal range of motion. They studied the trunk flexion/extension, trunk lateral flexion, and rotation (not including measures of cervical mobility) under supervised and unsupervised conditions; they also calculated a normalized index IMU-ASMI and evaluated its reliability. Franco et al. [33] evaluated five movements, trunk flexion, trunk extension, trunk lateral flexion, cervical rotation, and cervical flexion/extension, using five commercial inertial sensors. Gardiner et al. [34] evaluated the maximum range of motion at the cervical and lumbar spine with two ViMove brand inertial sensors. In previous works [35,36], the authors presented a preliminary test with only six sensors and evaluated flexion trunk and lateral flexion in two AS patients.

**Figure 2:**
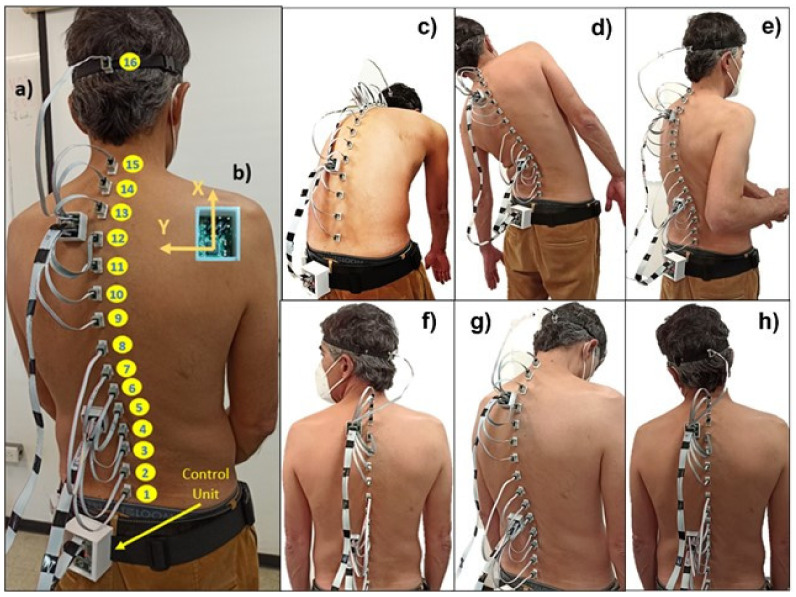
The embodiment of the system: (**a**) MIMUs over the patient’s spine; (**b**) frame reference of the sensors, with the *z*-axis out of the image. The movements considered in the experimental study: (**c**) anterior hip flexion; (**d**) trunk lateral flexions; (**e**) trunk axial rotation; (**f**) cervical axial rotation; (**g**) cervical flexion/extension; (**h**) cervical lateral flexion.

Even though the studies mentioned above provide accuracy and repeatability to the measurements, they are limited by the number of sensors they use and, therefore, the amount of information they can provide for subtle and complex movements. With this motivation, we developed a novel MIMU-based system for the evaluation of spine movements. The system consists of an array of 16 small MIMU units specifically designed to be placed along the entire spine (Figure 2a). We present the results of an experimental study aimed at validating the reliability and repeatability of the system. To the best of our knowledge, this is the only MIMU-based system capable of simultaneously monitoring different movements of the lumbar, thoracic, and cervical areas, considering the spine’s serial kinematic chain with multiple degrees of freedom in various configurations. Our results indicate that the system is reliable and feasible for clinical studies and applications. It paves the way for the design of better clinical evaluation methods, with objective metrics of spine mobility, especially in rheumatology and orthopedics.

## 2. Materials and Methods

### 2.1. Multi-MIMU System

The proposed system’s architecture consists of 16 small magnetic and inertial units (Invensense MPU-9250) and a wireless acquisition control unit. A printed circuit board was designed with the aim of having a sensor with small enough dimensions (11.7 mm × 9.3 mm) suitable for monitoring subtle movements without limiting mobility. In this way, an array of multiple sensors can be easily placed in the subjects’ back and head to provide kinetic information of the entire spine at the cervical, thoracic, and lumbar sites.

Each sensor contains a triaxial accelerometer, a triaxial gyroscope, and a triaxial magnetometer (±2 g range, ±250°/s range, ±4800 µT full range, respectively) to evaluate the subject’s movements in three dimensions. The signals of the sensors were recollected through a compact and portable wireless control unit mounted in the participant’s hip with a harness. The harness was designed to be comfortably worn in soft and hygienic material without limiting mobility during the assessment (Figure 2a).

**Figure 3:**
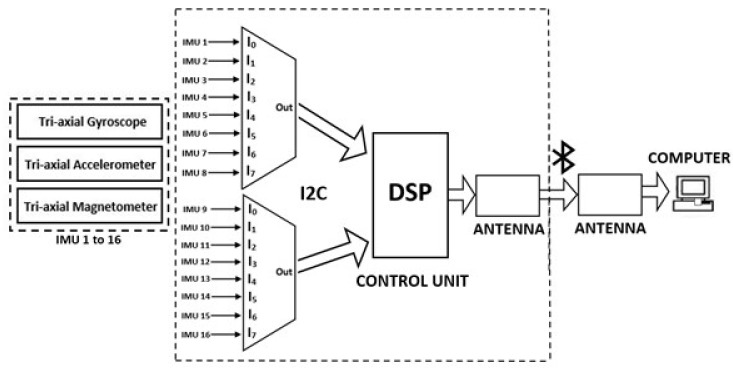
Architecture of the control unit of the proposed portable and wireless system.

The control unit consists of a digital signal processor (DSP-Teensy 3.2), interfaced with the sensors through two eight-channel fast multiplexors (TCA9548A) for I2C communication. The DSP collects the raw data from the sensors in real time and sends the information to a computer through a Bluetooth wireless communication protocol. A software desktop application was implemented to concurrently receive the raw data of the sensors and then estimate the orientation of each sensor, using a set of instances of a Kalman filter algorithm, with each instance specifically associated and calibrated per sensor [37]. The complete solution provides kinematic pose estimation of the participant’s spinal mobility at a frame rate of 20 Hz per sensor (Figure 3).

### 2.2. Movement Estimation Algorithm

The system comprises 16 MIMUs communicating wirelessly with a computer via a Bluetooth protocol. Each MIMU contains a triaxial accelerometer, gyroscope, and magnetometer. They are placed on different spine segments to evaluate movements in three dimensions. The tilt angles (roll and pitch) and the heading angle (yaw) are calculated through the numerical integration of the angular velocity provided by the gyroscope in the *y-*, *z-*, and *x*-axis according to the reference frame (Figure 2b). It is known that the gyroscopes present a drift problem due to the accumulative errors during the recursive calculus.

**Figure 4:**
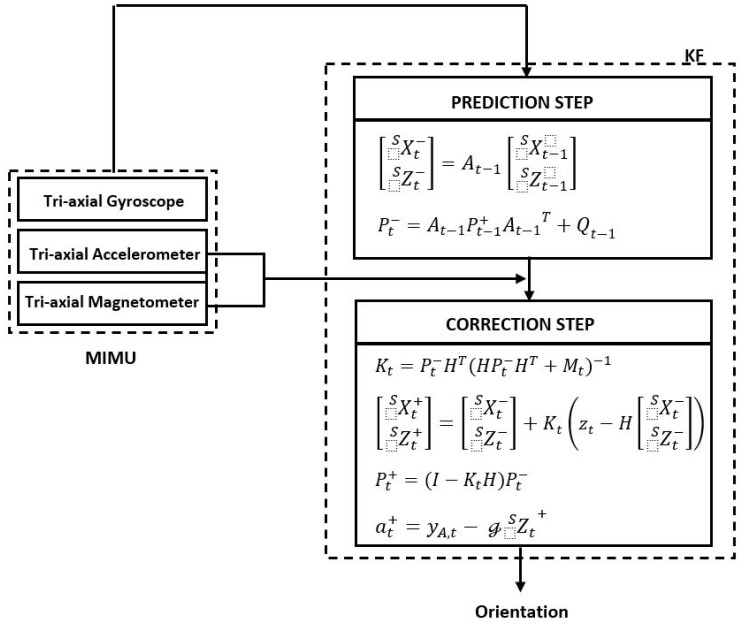
Chart of all the steps of the proposed Kalman filter algorithm, structured in two phases: the prediction and correction phases.

The problem is solved through the implementation of a sensor fusion of the gyroscope, accelerometer, and magnetometer signals using a Kalman filter (KF) [38,39,40]. With the KF, each MIMU orientation is estimated in two phases; it is first predicted using the gyroscope signals, and then corrected by the spatial references provided by the accelerometer and magnetometer signals. The accelerometers for the tilt angles and the magnetometers for the heading angle (Figure 4).

According to the KF proposed by Lee et al. [39] and Ligorio and Sabatini [40] for estimating the two tilt angles, we implemented a modified algorithm version to include the heading angle. The purpose is to estimate the vectors XS and ZS from the rotation matrix *R*, which allows the coordinate transformation from the sensor frame *S* to the inertial frame *I*. On the basis of the *ZYX* Euler angles, *R* is expressed as

(1) $$R=\left[\begin{array}{ccc} cos\alpha cos\beta & cos\alpha sin\beta sin\gamma -sin\alpha cos\gamma & cos\alpha sin\beta cos\gamma +sin\alpha sin\gamma \\ sin\alpha cos\beta & sin\alpha sin\beta sin\gamma +cos\alpha cos\gamma & sin\alpha sin\beta cos\gamma -cos\alpha sin\gamma \\ -sin\beta & cos\beta sin\gamma & cos\beta cos\gamma \end{array}\right],$$

where *α* is the yaw or heading angle, *β* is the pitch angle, and *γ* is the roll angle, known as tilt angles.

The vector ZS (i.e., the last row of the matrix *R*) in Equation (1) is expressed in terms of *γ* and *β*. Hence, knowing ZS, tilt angles can be calculated as follows:

(2) γ=tan−1ZS2ZS3 and β=tan−1ZS1ZS2/sinγ.

Similarly, knowing XS (i.e., the first row of the matrix *R*) and roll and pitch angles, heading angle *α* can be determined by

(3) α=tan−1−cosγXS2+sinγXS3XS1/cosβ.

#### 2.2.1. Sensor Modeling

The sensor signals from the gyroscope are modeled by

(4) $$y_{G}=\omega +n_{G},$$

where ω is the angular velocity, and $n_{G}\;$ is the measurement noise that is assumed to be zero-mean white Gaussian.

The signals from the accelerometer are modeled as

(5) $$y_{A}=g+a+n_{A}.\;$$

where g is the gravity vector with respect to the sensor frame, defined as ℊ×ZS where ℊ is $9.8\frac{m}{s^{2}\;}$, a is the external acceleration, and $n_{A}\;$is the measurement noise assumed as zero-mean white Gaussian. As established by Luinge et al. [41], in Equation (5), the external acceleration a can be modeled as a first-order low-pass filtered white noise process.

(6) $$a_{t}=c_{a}a_{t-1}+\varepsilon_{t},$$

where $c_{a}$, which determines the cutoff frequency, is a dimensionless constant between 0 and 1, and $\varepsilon_{t}\;$ is the time-varying error of the acceleration model.

Lastly, the signals from the magnetometers are modeled as

(7) $$y_{M}=h+n_{M},$$

setting h (the actual Earth’s magnetic field vector) as

(8) $$h=A^{-1}\left(h_{m}-b\right),$$

where $A^{-1}$ is the inverse of the soft-iron interference matrix, b is the hard-iron interference matrix, and $h_{m}$ is the distorted Earth’s magnetic field (i.e., in the presence of soft-iron and hard-iron interferences) [42]. As in the gyroscope and accelerometer signals model, $n_{M}$ is the measurement noise assumed as zero-mean white Gaussian. It is important to develop a previous characterization step of the sensors, to set the values of the measurement noises (i.e., $n_{G}$, $n_{A}$, and $n_{M}$), as well as the soft-iron and hard-iron interference matrices [37,43].

#### 2.2.2. Kalman Filter Design

The KF is defined by the following process model [44]:

(9) $$x_{t}=A_{t-1}x_{t-1}+w_{t-1},$$

and the following measurement model:

(10) $$z_{t}=Hx_{t}+v_{t},$$

where $x_{t}$ in Equation (9) is the state vector, defined as xt=XSt−ZSt−T, A is the state transition matrix that relates the state at a previous time step t−1 to the state at the current step t, and w is the white Gaussian process noise. In Equation (10), $z_{t}$ is the measurement vector, H is the observation matrix that relates the state (i.e., $x_{t}$) to the measurement $z_{t}$, and v is the white Gaussian measurement noise. The minus superscript in the state vector denotes the a priori estimate.

The orientation is a priori estimated by the integration of the gyroscope signals, giving the following process model:

(11) XSt−ZSt−=I+Δtω˜t−100ω˜t−1TXSt−1ZSt−1,

where Δt is the time interval and $\tilde{\omega }$ is the skew-symmetric matrix function of the vector ω, denoted as

(12) $$\tilde{\omega }=\left[\begin{array}{ccc} 0 & -\omega_{z} & \omega_{y}\\ \omega_{z} & 0 & -\omega_{x}\\ -\omega_{y} & \omega_{x} & 0 \end{array}\right].$$

Due to the noisy measurements of the angular velocity in Equation (4), Equation (9) must be expressed using the current gyroscope output (i.e., $y_{G}=\omega +n_{G}$) as

(13) XSt−ZSt−=I−Δty˜G,t−100y˜G,t−1XSt−1ZSt−1+Δtn˜G00n˜GXSt−1ZSt−1.

Following the mathematical deduction reported by Lee et al. [39], Equation (13) can be developed as

(14) XSt−ZSt−=I−Δty˜G,t−100y˜G,t−1XSt−1ZSt−1+Δt−X˜St−100−Z˜St−1nGnG.

Thus, from Equation (14), the transition matrix $A_{t-1}$ and the process noise $w_{t-1}$ can be defined as

(15) $$A_{t-1}=\left[I-\Delta t\left[\begin{array}{cc} {\tilde{y}}_{G,t-1} & 0\\ 0 & {\tilde{y}}_{G,t-1} \end{array}\right]\right],$$

(16) wt−1=·Δt−X˜St−100−Z˜St−1nGnG.

Then, the process noise covariance matrix $Q_{t-1}$ defined by $E\left[w_{t-1}w_{t-1}^{T}\right]$ can be redefined using Equation (16) as

(17) Qt−1=−Δt2X˜St−100Z˜St−1ΣGX˜St−100Z˜St−1,

where $\Sigma_{G}$, defined by $E\left[n_{G}n_{G}^{T}\right]$, is the covariance matrix of the gyroscope measurements noise and is established equal to

(18) $$\Sigma_{G}=\left[\begin{array}{cc} \sigma_{G}^{2} & 0\\ 0 & \sigma_{G}^{2} \end{array}\right].$$

In Equation (18), $\sigma_{G}^{2}$ is obtained in a previous characterization step and is a 3 × 3 matrix where the gyroscope noise variances of the *x-*, *y-*, and *z*-directions are in the main diagonal.

The measurement model is based on the accelerometer and magnetometer measurements since they give the spatial reference to correct the estimation error in the process model. As in [41], the error of the predicted acceleration is defined as

(19) $$a_{\varepsilon ,t}^{-}=a_{t}^{-}-a_{t},$$

where $a_{t}^{-}$ is the a priori estimate of the external acceleration of the current time step and is defined as $c_{a}a_{t-1}^{+}$, which is available from a previous time. Note that the plus superscript denotes the a posteriori estimate after the filter correction.

Therefore, using Equations (6) and (19), Equation (5) can be rewritten as

(20) yA,t−caat−1+=ℊZSt−aε,t−+nA.

From Equation (7), h, the actual Earth’s magnetic field vector, is

(21) h=𝒽XS,

where 𝒽 is the value of the local Earth’s magnetic field. Hence, from Equations (20) and (21), the measurement model can be established sd follows:

(22) yM,tyA,t−caat−1+=𝒽I300ℊI3XSt−ZSt−+nM−aε,t−+nA.

Accordingly, the measurement vector $z_{t}$, the observation matrix H, and the measurement noise $v_{t}$ are

(23) $$z_{t}=\left[\begin{array}{c} y_{M,t}\\ y_{A,t}-c_{a}a_{t-1}^{+} \end{array}\right],$$

(24) $$H=\left[\begin{array}{cc} 𝒽I_{3} & 0\\ 0 & ℊI_{3} \end{array}\right],$$

(25) $$v_{t}=\left[\begin{array}{c} n_{M}\\ -a_{\varepsilon ,t}^{-}+n_{A} \end{array}\right].$$

Since $a_{\varepsilon ,t}^{-}$, $n_{A},\;$ and $n_{M}$ are uncorrelated, the measurement noise covariance matrix, $M_{t}$, defined by $E\left[v_{t}v_{t}^{T}\right]$, results in

(26) $$M_{t}=\left[\begin{array}{cc} \Sigma_{M} & 0\\ 0 & \Sigma_{acc}+\Sigma_{A} \end{array}\right],$$

where $\Sigma_{M}$, $\Sigma_{acc}$, and $\Sigma_{A}$ are the covariance matrices of the magnetometer measurement noise, the acceleration model error, and the accelerometer measurement noise, respectively. $\Sigma_{M}$ is defined as $\sigma_{M}^{2}$, which is the 3 × 3 matrix where the magnetometer noise variances of *XYZ* directions are in the main diagonal. As in [39], $\Sigma_{acc}$ is defined as $3^{-1}c_{a}^{2}\|a_{t-1}^{+}{\|}^{2}I$, where $\|a_{t-1}^{+}{\|}^{2}$ is the square of the vector norm, and $\Sigma_{A}$ is set as $\sigma_{A}^{2}$, the 3 × 3 matrix where the accelerometer noise variances of *XYZ* directions are in the main diagonal.

Eventually, once XSt−ZSt−T are estimated, the external acceleration $a_{t}^{+}$ can be calculated by

(27) at+=yA,t−ℊZSt+.

### 2.3. Experimental Study

#### 2.3.1. The Objective of the Study

The objective of the study was to assess the reliability and repeatability of the proposed system for the clinical evaluation of spine motility. To this end, six movements were considered: three movements involving the articulations along the thoracic and lumbar spine (anterior hip flexion, trunk lateral flexions, and trunk axial rotation) and three involving the cervical spine (cervical axial rotation, cervical flexion/extension, and cervical lateral flexion).

#### 2.3.2. Recruitment

Fifteen healthy participants were invited to participate in the study (eight male and seven female). The inclusion criteria were being young adults without any mobility impairment, history of diagnosis of spine or musculoskeletal ailment, or presence of any joint or spinal pain at the time of the study. The individuals shared homogeneous anthropometric characteristics such as weight, height, and body mass index (BMI) to guarantee the same physical mobility capabilities among participants. The study was approved by the Local Ethics Committee of the General Hospital of Mexico “Dr. Eduardo Liceaga” (protocol code DI/03/17/471). All participants signed the informed consent form.

#### 2.3.3. Experimental Procedure

The participants performed two repeated exercise sequences during complete sessions on 2 days each, 1 week apart from the first to the second sequence. The sessions were carried out at approximately the same period during the day on mornings. All experiments and recordings were conducted by the same operator to assess the repeatability of the system.

Sociodemographic data and anthropometric measurements were collected on the first day of the study. Each day, every subject was equipped with 16 sensors along the spine, one on the head occiput to assess the cervical movements, and 15 along the back. The first back sensor was attached over the first sacral vertebra (S1) and the last was attached over the last cervical vertebra (C7), leaving an equidistant separation between each sensor. The distance between sensors was different on each subject depending on their height and natural erect posture (Figure 2a). The back sensors were mounted directly on the skin with double-sided tape, and the head sensor was mounted with a strap.

The spinal assessment protocol included six movements (Figure 2c–h), including three from the original BASMI method: cervical rotation, anterior hip flexion, and trunk side flexion. Instead of tragus-to-wall distance, we propose introducing two other head movements, the cervical flexion/extension and cervical lateral flexion. Additionally, we studied the trunk axial rotation along the lumbar and thoracic spine.

- *Movement 1*: anterior hip flexion.
- *Movement 2*: trunk lateral flexions to the left/right sides.
- *Movement 3*: trunk axial rotation.
- *Movement 4*: cervical axial rotation.
- *Movement 5*: cervical flexion/extension.
- *Movement 6*: cervical lateral flexion.

The operator kindly guided the participants using standardized instructions through each movement. Participants were asked to perform their maximum effort without feeling any pain or discomfort to reach their full range of motion (ROM). Each movement was repeated three times at a slow and constant speed; before starting every series, the participants stood in a natural upright position with their feet shoulder-width apart for 5 s to set the baseline position of each sensor. All participants performed the sequence of six-movement repetitions strictly in the same order to prevent any possible bias due to arbitrary execution of the exercise routines.

#### 2.3.4. Measurements

For all subjects, the Euler angles in *ZYX* representation were simultaneously estimated for each MIMU for all six movement series. For movement 1, movement 2, and movement 3, 15 sensor units were considered, from MIMU1 to MIMU15, placed approximately over the S1 sacral vertebra up to the C7 cervical vertebra. Thus, there were seven sensor units (MIMUs 1–7) for the lumbar spine and eight units (MIMUs 8–15) for the thoracic spine. For movement 4, movement 5, and movement 6, three sensor units were considered, from MIMU14 to MIMU16, with MIMU14 between thoracic vertebrae T1 and T2, MIMU15 at the C7 vertebra, and MIMU16 over the occipital area of the head. Then, the ranges of motion (RoM) of frontal flexions of movement 1 and cervival flexions/extensions of movement 5 were estimated from the maximum and minimum roll angles of every sensor unit. The RoM of lateral flexions of movement 2 and movement 6 were estimated from maximum and minimum pitch angles; RoM of axial rotations of movement 3 and movement 4 were estimated analogously from yaw angles.

#### 2.3.5. Statistical Analysis

The uniformity of the age and anthropometric measurements was statistically evaluated using the *t*-test. Descriptive statistics (mean, SD, and ranges) over the RoM of the participants per MIMU, grouping for the first and second measurements, were computed.

To analyze the repeatability of the system. The intraclass correlation coefficient (ICC) was first computed for each MIMU, for each movement separately from the mean, for different pairs of measurements. Then, the grand ICC was estimated per movement, grouping the pairs of measurements of all the corresponding MIMUs involved in the respective exercise. ICC interpretation, based on [45], was as follows: <0.4 = poor, 0.41 to 0.6 = regular, 0.61 to 0.8 = good, and >0.81 = excellent. A significance level of *p* < 0.05 was assumed for all statistical tests. The statistical tests and analysis were carried out in Matlab R19 and SPSS 20.

## 3. Results

The study group comprised 15 healthy participants. A one-sample Student’s *t*-test (two-tailed version) confirmed the homogeneity of participants’ anthropometric characteristics, resulting in significant descriptive statistics for all the metrics, as shown in Table 1. Thirteen participants completed the entire study. Data from two participants were discarded for analysis because they had issues with the fall of some sensors.

### Spinal Mobility Data

Descriptive statistics of the RoM for each MIMU and every movement are reported as angles in Table 2. The RoM obtained for every section of the spine was consistent with that reported in [46,47,48].

The reliability per sensor was assessed, in general showing good to excellent correlation in movements 1, 2, 4, 5, and 6 for most MIMUs.

**Figure 5:**
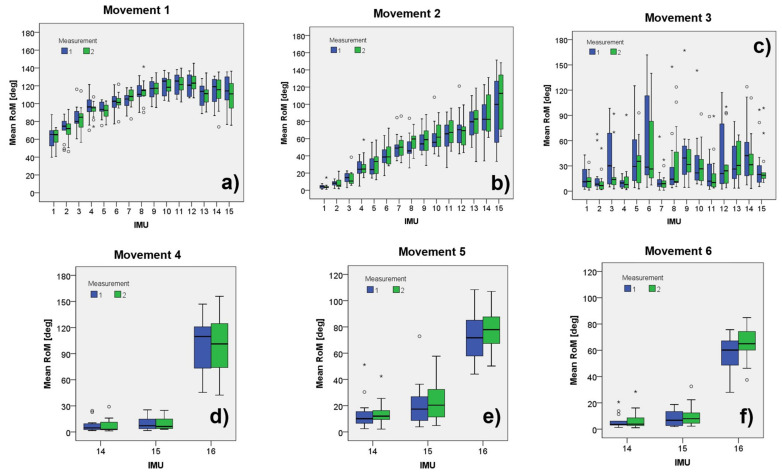
Box plots showing the distribution of the observed RoM with the different MIMUs placed along the subjects’ back and head for the six movements: (**a**) anterior hip flexions; (**b**) trunk lateral flexions; (**c**) trunk axial rotations; (**d**) cervical axial rotation; (**e**) cervical flexion/extension; (**f**) cervical lateral flexions. The blue bars correspond to the first series of measurements, while the green ones correspond to the second series of measures, * and ° indicate observed outliers.

Figure 5 presents bar plots showing the medians, ranges, and outliers obtained from each combination of 16 IMU × 2 measurements × 6 movements.

**Figure 6:**
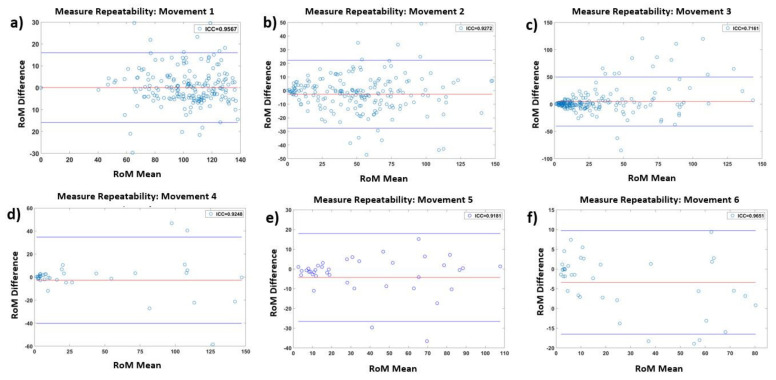
Bland–Altman plots presenting the mean difference plots showing the agreement between the two repeated measurements of the ranges of motion of the inertial measurement units placed along the subjects’ back and head for the six movements: (**a**) anterior hip flexions; (**b**) trunk lateral flexions; (**c**) trunk axial rotations; (**d**) cervical axial rotation; (**e**) cervical flexion/extension; (**f**) cervical lateral flexions.

Bland–Altman plots in Figure 6 show the agreements of the RoM for every movement. As can be observed, the system presents good agreement for five of the six studied exercises, with few observed outliers, except for trunk axial rotation (Figure 6c).

Table 3 presents the detailed descriptive statistics of the corresponding Bland–Altman plots in Figure 6, confirming the high repeatability for frontal and lateral flexions of the lumbar–thoracic spine and the cervical spine in axial rotation, flexion/extension, and lateral flexions. Additionally, it shows good repeatability for the axial rotation of the trunk.

## 4. Discussion

The purpose of this study was to demonstrate the reliability and repeatability of the proposed novel system. The system comprises 16 miniaturized sensors to assess the entire spine, including the cervical, thoracic, and lumbar sections, unlike previous studies [31,32,34] which used only two sensors to evaluate the cervical spine and two sensors to assess the lumbar spine in separate tests. This configuration with more sensors allows obtaining more motion information at the highest spatial resolution than existing approaches. For this reason, it is expected that the system would provide metrics more sensitive to changes in the progress of the impairment or improvements due to treatments in patients with musculoskeletal disorders, especially disabilities due to AS.

The intrarater reliability analysis for all the MIMUs (Table 2) showed mostly a good (0.61 < ICCs < 0.8) to excellent (>0.81) intraclass correlation coefficient. In particular, for the anterior hip flexion exercise (Movement 1), seven of the 15 used sensors presented a good level of reliability, as exhibited by the achieved ICC, six presented an excellent reliability level, and only two presented a moderate level. These two were placed at the bottom part of the lumbar spine.

For the trunk lateral flexion exercise (Movement 2), for the same 16 sensors, the system presented 10 sensors with a good intraclass correlation coefficient but five sensors with poor coefficients, three of them located in the lumbar region. The observed reliability level of the three sensors placed at the lumbar site showed similar behavior to the coefficient reported in [34] of the sensor located in the same anatomical region. Sensors 1 to 5 showed a poor ICC due to their position on the participants’ back. For MIMU 1, a possible explanation is that it was too close to the participants’ clothes, which may have induced erratic sliding of the sensor during the exercises. For sensors 2–5, the observed variability in the lumbar ICCs was likely due to anatomical factors rather than sensor error. Because participants have different body compositions (adipose tissue), a narrower channel is generated in the center of the back between the muscles, making it difficult to place the sensors and keep them in place without undesired sliding motions given the skin displacements.

For trunk axial rotation (Movement 3), the system did not give helpful information, at least not for the entire spine, in the tested configuration. The ICCs were poor in almost all sensors, except four of five sensors placed on the upper thoracic spine (MIMU 11–15). A suitable explanation is that the localization of the sensors was not optimal in the selected configuration. In other words, placing them along the spine also causes erratic sliding motions of the sensors due to skin deformation during the exercises. Furthermore, a signal-to-noise problem is plausible due to the noise increment with the erratic movements of the sensors. Thus, it is necessary to continue researching the best configuration to place the sensors for axial rotation. The design presented by Molnar et al. [30] could be a better option since the sensors were placed on both sides of the spine at sites with less skin displacement.

Concerning the exercises involving cervical movements (Movements 4–6), sensor 16 was attached with a strap to the head occiput. This sensor measures the range of motion of the cervical spine with respect to the back. Variability in the cervical spine ranges of motion was observed among participants, resulting in good but not excellent reliability. We suspect that not all the participants performed their maximum ranges of motion in trying to complete the series dynamically, as we were able to visually observe during the experiments. Personal natural anatomical limitations despite the anthropometric homogeneity of the population cannot be ruled out either. An option could be to record the ranges of motion in static conditions, ensuring that the maximum range has been reached, providing consistency in measurements. In any case, the dynamic recording of the exercises could give valuable temporal and differential information for future research.

As can be observed, some factors influenced the variability of the system, some attributed to the population itself, and some attributed to measurement errors. However, following the overall intraclass correlation analysis to assess the global intraobserver reliability of the system (Table 3), an excellent level of intraclass correlation was evidenced for five of the six considered movements (i.e., Movements 1, 2, 4, 5, and 6), whereas one had good ICC (Movement 3). These results indicate acceptable repeatability of the system and, thus, feasibility for further clinical studies.

From the results of the intrarater variability, it is deduced that the system offers advantages over manual clinical methods (BASMI) because it can provide the clinicians with more relevant information on how the patient performs movements, as well as how it is affected by the clinical condition, in terms of speed, smoothness of movements, continuity, and fluidity, in a more systematic and controlled fashion, thereby reducing subjectivity.

As a limitation of the current study, for the moment, we evaluated the repeatability of the system in static conditions considering the evaluation of the RoM; however, it would be interesting and require extensive research effort to assess the reliability of the system regarding the dynamic execution of the movements. This may be valuable for introducing new metrics involving temporal patterns, movements phases, and abnormal motion synergies not considered in current clinical practice.

Thus, as future work, an information reduction analysis can be done to obtain a more efficient system, with only the necessary sensors and correct locations maintained for providing important information and facilitating its practical use in clinical environments. Furthermore, the design of an ergonomic suit and belt is needed. In any case, our proposed system features the largest number of wearable sensors, allowing the investigation of new and different configurations. This can be helpful to evaluate not only the spine but also other joints such as the arms, legs, and fingers, which can also be affected by AS (or other musculoskeletal conditions). Moreover, further studies need to be conducted to confirm the inter-rater reliability involving at least two different observers.

Regarding the validity and accuracy of the system, the registered ranges of motion are consistent with the literature on spinal biomechanics [46,47,48] and similar to those reported by Franco et al. [33]. Even so, further study is needed to assess the accuracy of the system. Additionally, the study was conducted in a controlled environment where electromagnetic noise was characterized. However, for its implementation in clinical practice, it is necessary to enhance the Kalman filter to neutralize uncontrolled electromagnetic noise that may not be adequately characterized, similarly to the measurement noise covariance reported in [49,50]. Once again, more studies testing the repeatability of the instruments should be considered in the future.

## 5. Conclusions

This paper presented the development and evaluation of a novel magnetic and inertial measurement unit-based system to assess the entire spine’s motion. The system comprises 16 MIMUs unlike previous studies that used at most five sensors. The applied protocol demonstrated the reliability of using the system to evaluate the entire spine, with an excellent level of intraclass correlation (>0.81) for five of the six considered movements. The present study introduced three movements (i.e., trunk rotation, cervical flexion/extension, and cervical lateral flexion), unfeasible to evaluate using manual methods such as the BASMI, the most used index for spine assessment in AS. However, it is necessary to continue researching the best configuration to place the sensors for axial rotation since the proposed approach did not give helpful information. According to the tests performed, spine evaluation using the proposed approach presents advantages over traditional methods. In addition, the quantity and configuration of the MIMUs allow obtaining more objective information on the patient’s condition for a better and more timely diagnosis. The system represents a valuable tool to investigate better and more accurate evaluation metrics than current clinical methods.

## Figures and Tables

**Table 1:** The participants’ anthropometric information shows a uniform population, indicating similar physical conditions and capacities for performing the spine and head movements.

Subjects Anthropometric Data		
Participants		
Women	7	
Men	8	
	mean ± SD	*p* value (*t*-test)
Age	31.26 ± 5.79	<0.0001
Weight (kg)	67.15 ± 10.18	<0.0001
Height (m)	1.67 ± 0.07	<0.0001
BMI (kg/m^2^)	24.04 ± 2.43	<0.0001
Back height (cm)	49.39 ± 3.02	<0.0001
Separation between MIMU (cm)	3.52 ± 0.21	<0.0001

**Table 2:** Descriptive statistics and intraclass correlation of registered ranges of motion by the MIMUs for the trunk and head movements, considering two repeated series of three movements by the same operator.

	Trunk Movements										Head Movements								
	Movement 1			Movement 2			Movement 3				Movement 4			Movement 5			Movement 6		
IMU	Mean	SD	ICC	Mean	SD	ICC	Mean	SD	ICC	IMU	Mean	SD	ICC	Mean	SD	ICC	Mean	SD	ICC
1	61.8	12.0	0.60	4.4	2.9	0.26	13.5	10.8	0.59	14	7.3	7.4	**0.84**	14.0	11.2	**0.92**	7.3	7.4	**0.86**
2	69.0	12.8	0.52	9.2	6.8	0.22	12.8	16.7	0.44	15	9.1	7.3	**0.87**	24.7	17.1	**0.82**	10.9	8.7	**0.71**
3	84.7	15.1	**0.91**	14.1	8.4	0.45	29.8	29.9	0.54	16	96.1	35.3	**0.67**	73.8	18.8	**0.72**	60.8	12.5	**0.66**
4	95.2	11.8	**0.63**	26.0	11.6	**0.71**	11.9	13.8	0.01										
5	92.7	8.7	**0.83**	28.8	13.7	0.57	38.5	32.6	0.38										
6	102.0	10.6	**0.85**	40.7	14.1	**0.67**	55.6	50.5	**0.84**										
7	105.8	9.7	**0.74**	49.6	15.4	**0.74**	11.7	13.0	0.03										
8	113.6	12.7	**0.82**	53.2	18.1	0.37	36.0	41.8	**0.84**										
9	116.5	11.7	**0.80**	59.4	23.6	**0.77**	38.7	28.4	0.45										
10	120.8	11.7	**0.77**	62.9	20.0	**0.67**	31.3	29.5	0.43										
11	123.8	12.3	**0.82**	66.2	19.6	**0.81**	19.1	23.7	**0.87**										
12	123.8	11.9	**0.73**	68.0	21.0	**0.65**	34.3	36.0	**0.77**										
13	110.1	13.6	**0.77**	81.6	27.9	**0.68**	37.6	25.4	**0.90**										
14	114.0	15.7	**0.84**	86.3	29.0	**0.70**	38.8	32.2	0.46										
15	112.8	17.7	**0.79**	102.3	40.7	**0.79**	26.5	26.2	**0.90**										

ICC scores highlighted in bold indicate a good to excellent level of reproducibility of the corresponding MIMU measurement.

**Table 3:** Overall intraclass correlation analysis to assess the repeatability of the system for the six considered movements.

Movement	Articulations	Excercise	IMUs	ICC
1	Lumbar–thoracic	Anterior hip flexion	MIMU1–MIMU15	**0.96**
2	Lumbar–thoracic	Lateral flexion	MIMU1–MIMU15	**0.93**
3	Lumbar–thoracic	Axial rotation	MIMU1–MIMU15	0.72
4	Cervical	Axial rotation	MIMU14–MIMU16	**0.93**
5	Cervical	Flexion/extension	MIMU14–MIMU16	**0.92**
6	Cervical	Lateral flexion	MIMU14–MIMU16	**0.97**

ICC scores highlighted in bold indicate an excellent level of reproducibility of the system for the corresponding movement.
